# Supplementary material for: Inactivation of γ‐secretases leads to accumulation of substrates and non‐Alzheimer neurodegeneration
Source: EMBO Mol Med. 2017 Jun 6;9(8):1088–99. doi: 10.15252/emmm.201707561 (PMC5538297; doi:10.15252/emmm.201707561)
Supplement: Supplementary file 1 — Expanded View Figures PDF [file EMMM-9-1088-s001.pdf]

Expanded View Figures

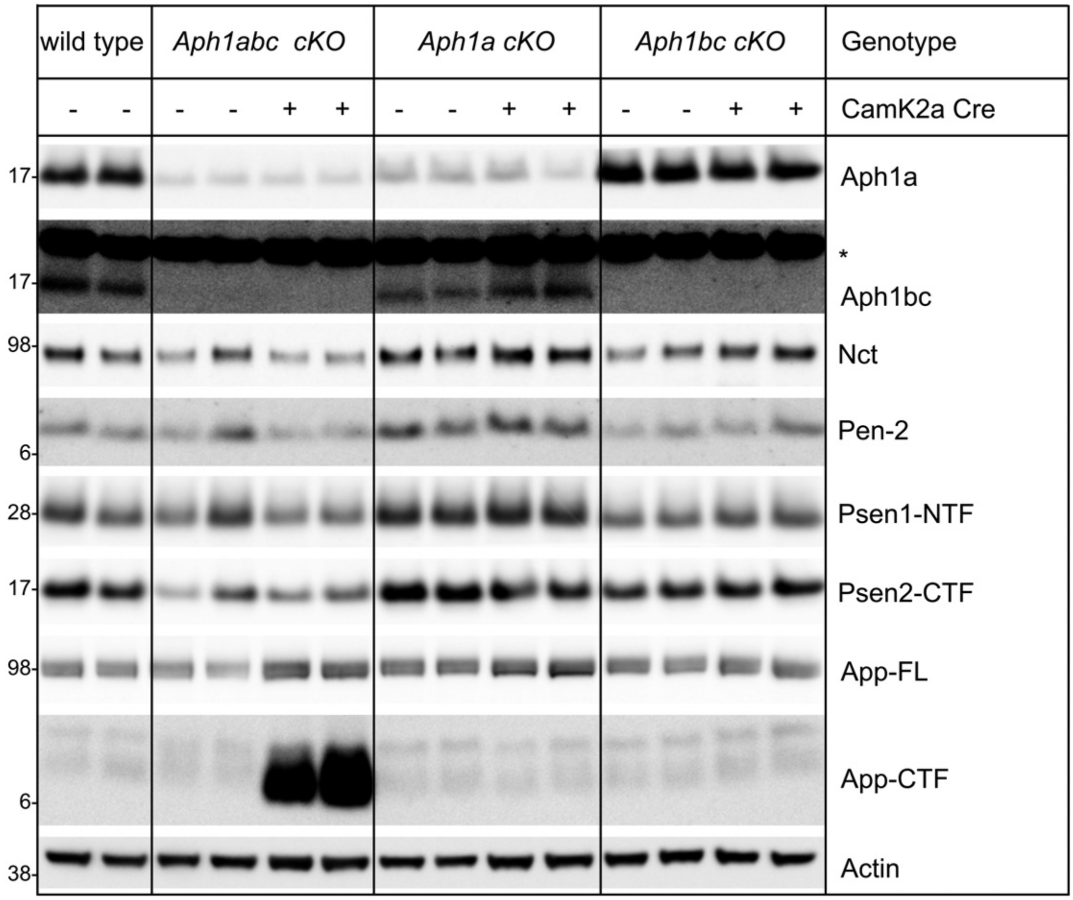

Figure EV1. Western blot analysis of cortical lysates of the different mouse strains used in the experiments.
Cortical lysates from six wild-type, six *Aph1abc cKO Cre*$^{-}$, six *Aph1abc cKO Cre*$^{+}$, six *Aph1a cKO Cre*$^{-}$, six *Aph1a cKO Cre*$^{+}$, six *Aph1bc cKO Cre*$^{-}$, and six *Aph1bc cKO Cre*$^{+}$ mice were analyzed by immunoblotting using antibodies against Nct, Pen-2, Aph1a, Aph1bc, Psen1-NTF, and App C-terminus. Two samples per genotype are shown. The asterisk indicates a non-specific band.

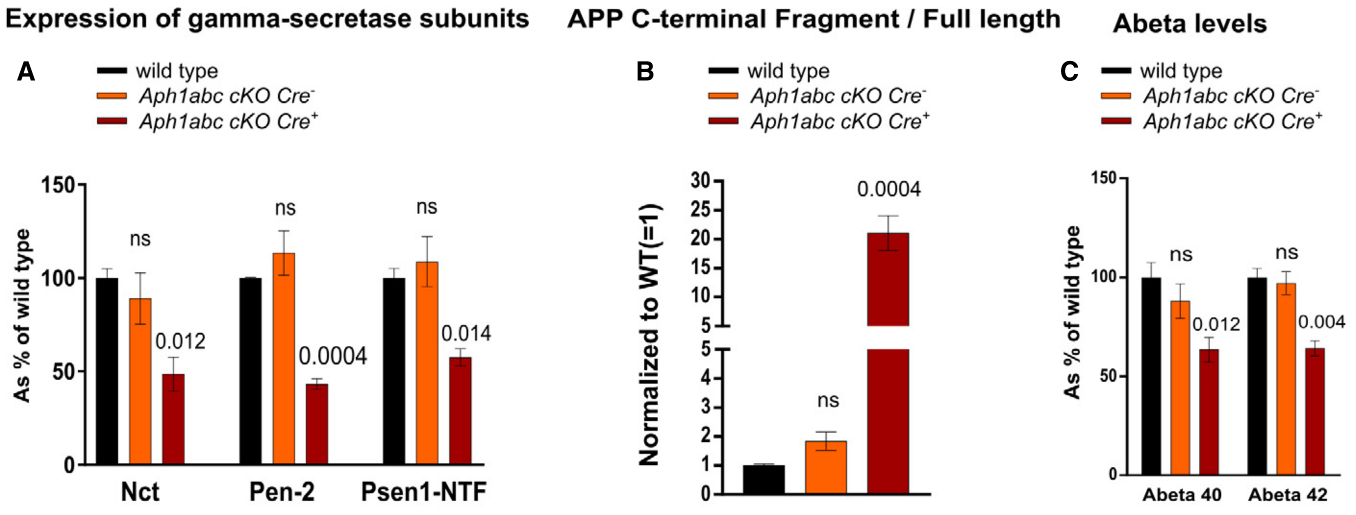

Figure EV2. Only complete loss of the Aph1 subunits in CaMKIIa neurons leads to decreased complex formation and impaired APP processing.

A Cortical lysates from wild-type, *Aph1abc cKO Cre*$^{-}$, and *Aph1abc cKO Cre*$^{+}$ were analyzed by immunoblotting using antibodies against Nct, Pen-2, and Psen1 NTF. Protein levels were standardized with β-actin and normalized to wild types. Mean, SEM, and *P*-values of 6 animals per genotype are shown. Differences in protein expression between wild-type and *Aph1abc cKO Cre*$^{-}$ or *Aph1abc cKO Cre*$^{+}$ were computed using one-way ANOVA per protein, followed by FDR *P*-value adjustment of a Dunnett's *post hoc* test.

B Cortical lysates from wild-type, *Aph1abc cKO Cre*$^{-}$, and *Aph1abc cKO Cre*$^{+}$ were analyzed by immunoblotting using antibodies against App C-terminus. Protein levels were quantified and FL/CTF ratios are plotted normalized to wild-type controls. Mean, SEM, and *P*-values of six animals per genotype are shown. One-way ANOVA per protein, followed by FDR *P*-value adjustment of the *P*-values obtained from a Dunnett's *post hoc* test.

C Cortical lysates from seven wild-type, seven *Aph1abc cKO Cre*$^{-}$, and seven *Aph1abc cKO Cre*$^{+}$ mice were analyzed by Aβ ELISA. Aβ levels were normalized to wild types. Mean, SEM, and *P*-values of seven animals are shown. One-way ANOVA per protein, followed by FDR *P*-value adjustment of a Dunnett's *post hoc* test. ns = not statistically significant.

Source data are available online for this figure.

 © 2017 The Authors

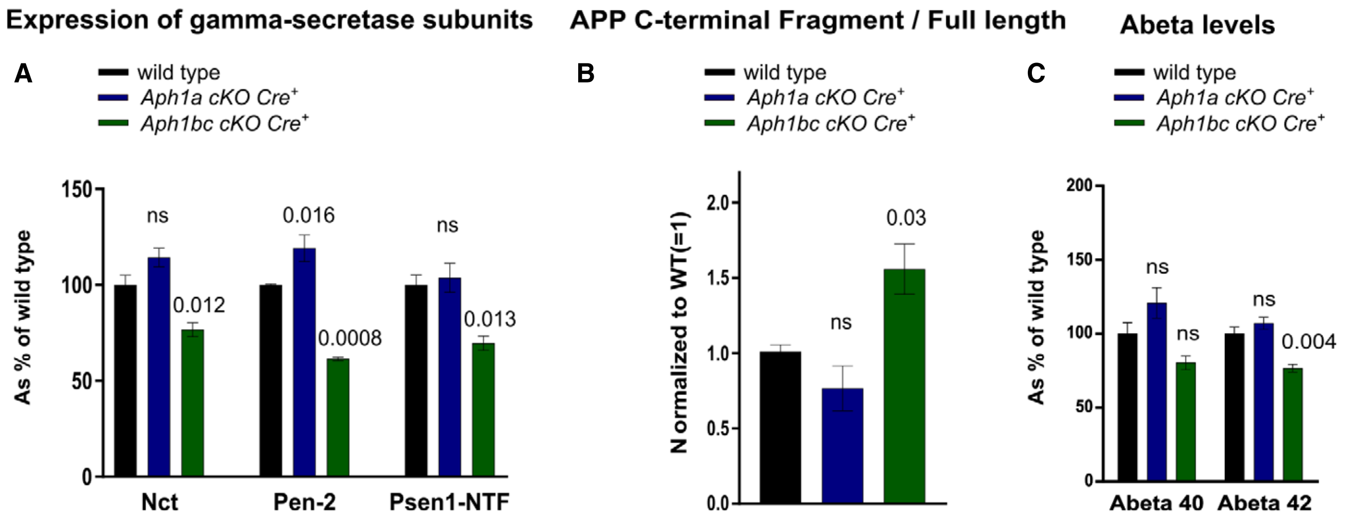

Figure EV3. Aph1bc is functionally more prominent with regard to reconstitution of mature complex.

A Cortical lysates from six wild-type, six *Aph1a cKO Cre*$^+$, and six *Aph1bc cKO Cre*$^+$ mice were analyzed by immunoblotting using antibodies against Nct, Pen-2 and Psen1-NTF. Protein levels were standardized with β-actin and normalized to wild type.

B Cortical lysates from six wild-type, six *Aph1a cKO Cre*$^+$, and six *Aph1bc cKO Cre*$^+$ mice were analyzed by immunoblotting using antibodies against App C-terminus. Protein levels were quantified and FL/CTF ratios are plotted normalized to wild-type control.

C Cortical lysates from seven wild-type, seven *Aph1a cKO Cre*$^+$, and seven *Aph1bc cKO Cre*$^+$ mice were analyzed by Aβ ELISA. Aβ levels were normalized to wild type.

Data information: Mean, SEM, and *P*-values are shown. One-way ANOVA per protein, followed by FDR *P*-value adjustment of a Dunnett's *post hoc* test. ns = not statistically significant.

Source data are available online for this figure.

© 2017 The Authors

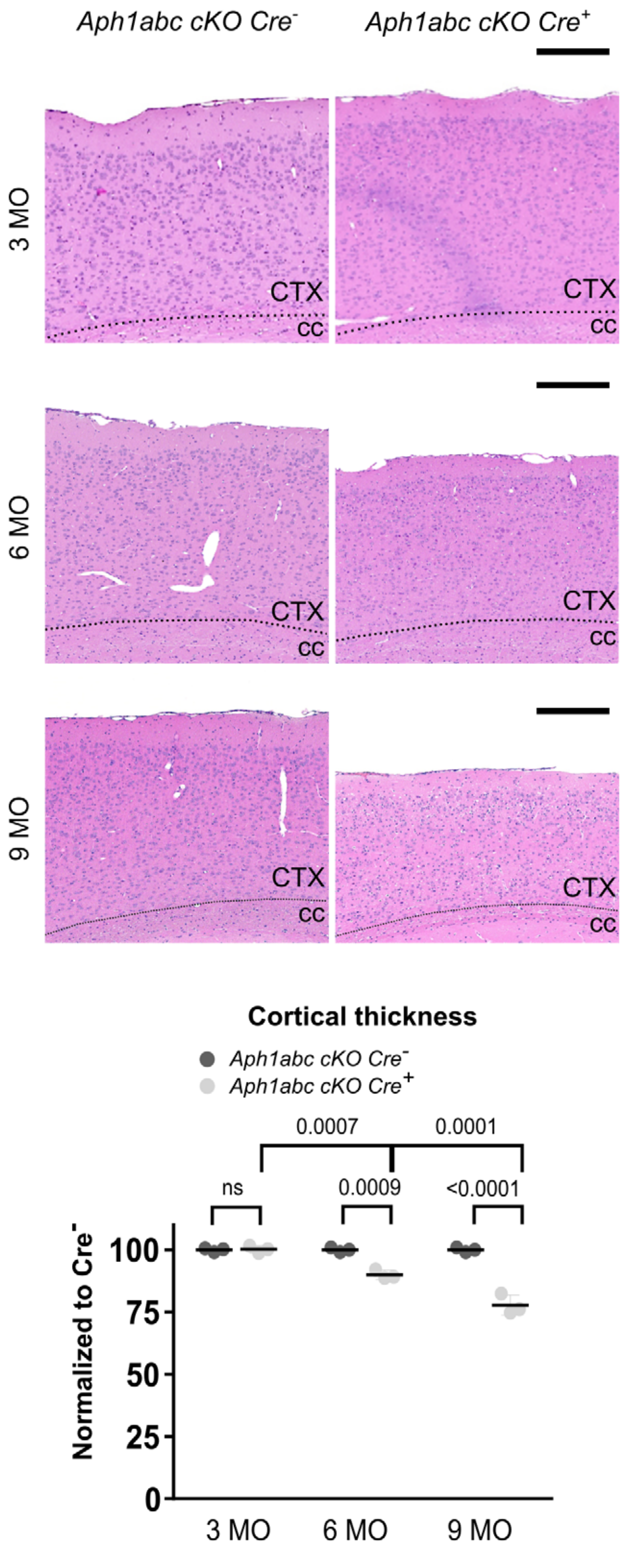

Figure EV4. Progressive neurodegenerative phenotype in *Aph1abc cKO Cre*+ mice.

Hematoxylin/eosin staining of sagittal brain slices of 3-, 6-, and 9-month-old mice. Scale bar = 195 μm. Absolute numbers of cortical thickness are normalized to *Cre*− littermates for each age. Progressive cortical atrophy is present in *Aph1abc cKO Cre*+ mice. Representative pictures for the 9-month-old mice have been reported also in Fig 1. Mean, standard deviation, and *P*-values are shown. Two-way ANOVA and Tukey's *post hoc* test. CTX = cortex; cc = corpus callosum, ns = not statistically significant. $N = 3$.

 © 2017 The Authors
