# Supplementary figures and images for: Inactivation of γ‐secretases leads to accumulation of substrates and non‐Alzheimer neurodegeneration
Source: EMBO Mol Med. 2017 Jun 6;9(8):1088–99. doi: 10.15252/emmm.201707561 (PMC5538297; doi:10.15252/emmm.201707561)

Figure EV2A, EV2B and Figure EV3A, EV3B

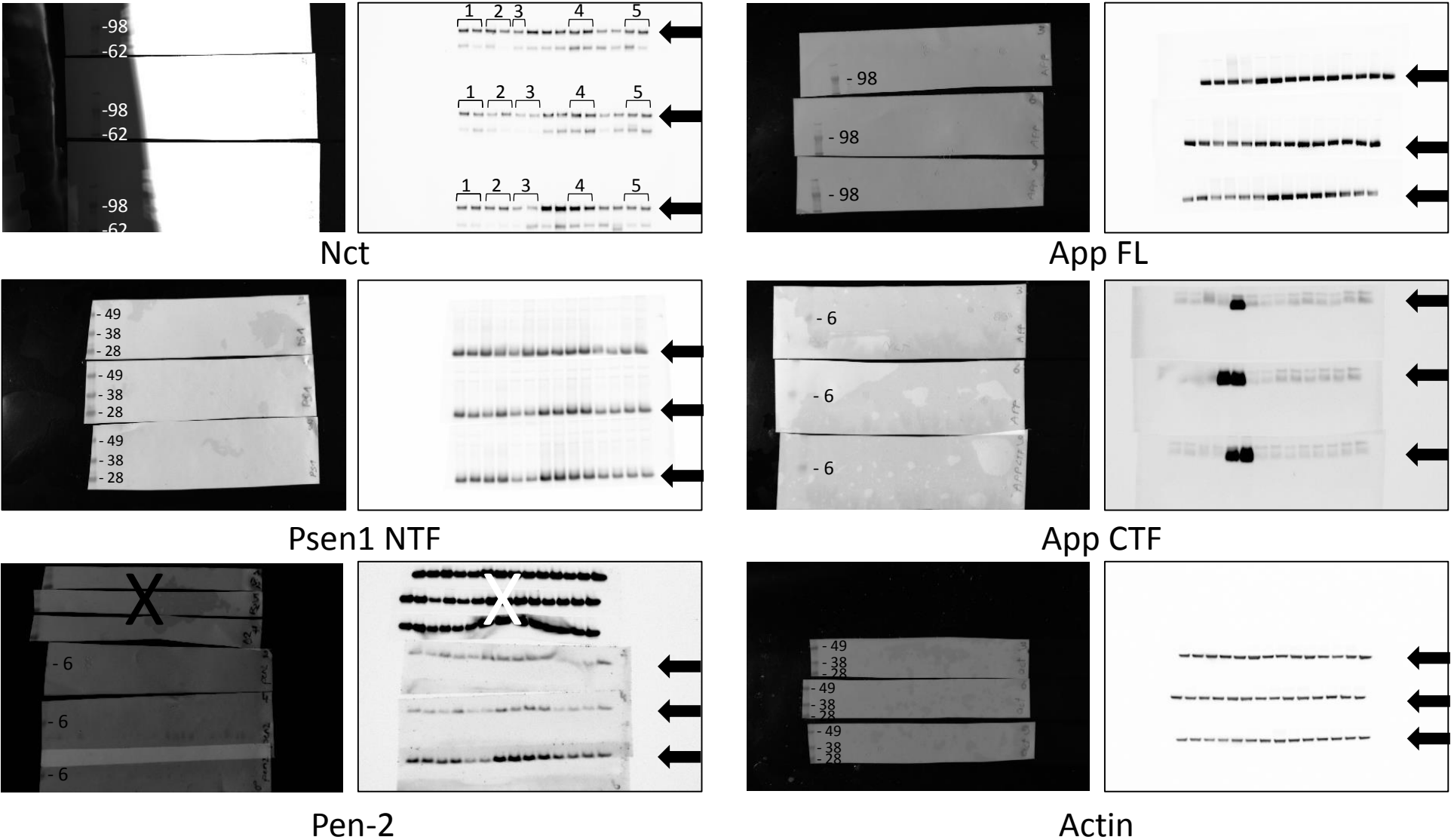

Supplement: Supplementary file 2 — Source Data for Expanded View [file EMMM-9-1088-s003.zip › Source_Data_for_EV_Figures/Source_Data_for_EV_Figures/Source_Data_for_FigsEV2andEV3.pdf]
