# Supplementary material for: Inactivation of γ‐secretases leads to accumulation of substrates and non‐Alzheimer neurodegeneration
Source: EMBO Mol Med. 2017 Jun 6;9(8):1088–99. doi: 10.15252/emmm.201707561 (PMC5538297; doi:10.15252/emmm.201707561)
Supplement: Supplementary file 4 — Source Data for Figure 3 [file EMMM-9-1088-s002.pdf]

Figure 3A

N = 5

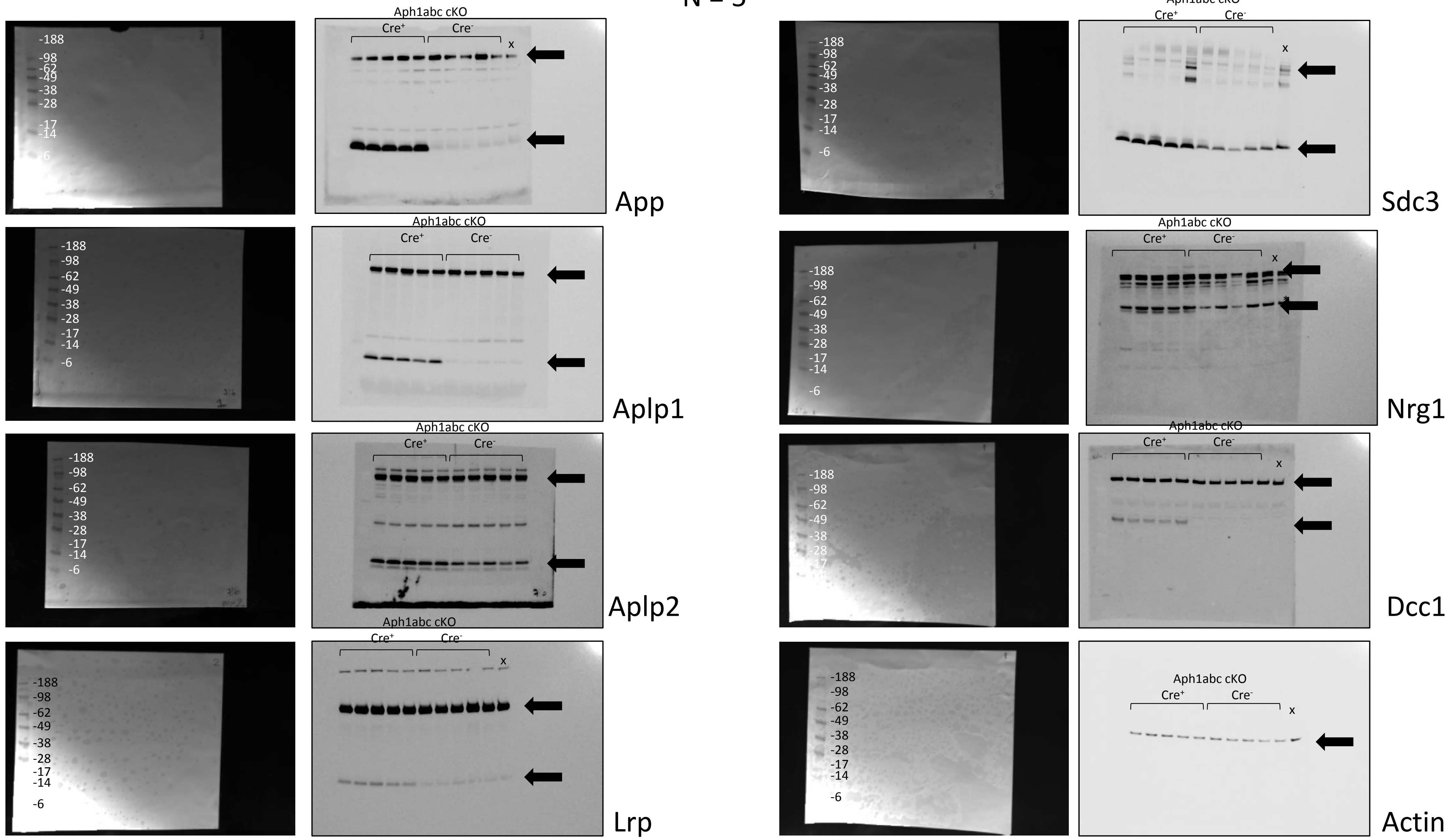
Aph1abc cKO
Cre+
Cre-
x
-188
-98
-62
-49
-38
-28
-17
-14
-6
App
Aplp1
Aplp2
Lrp
Sdc3
Nrg1
Dcc1
Actin

Figure 3B

N = 10

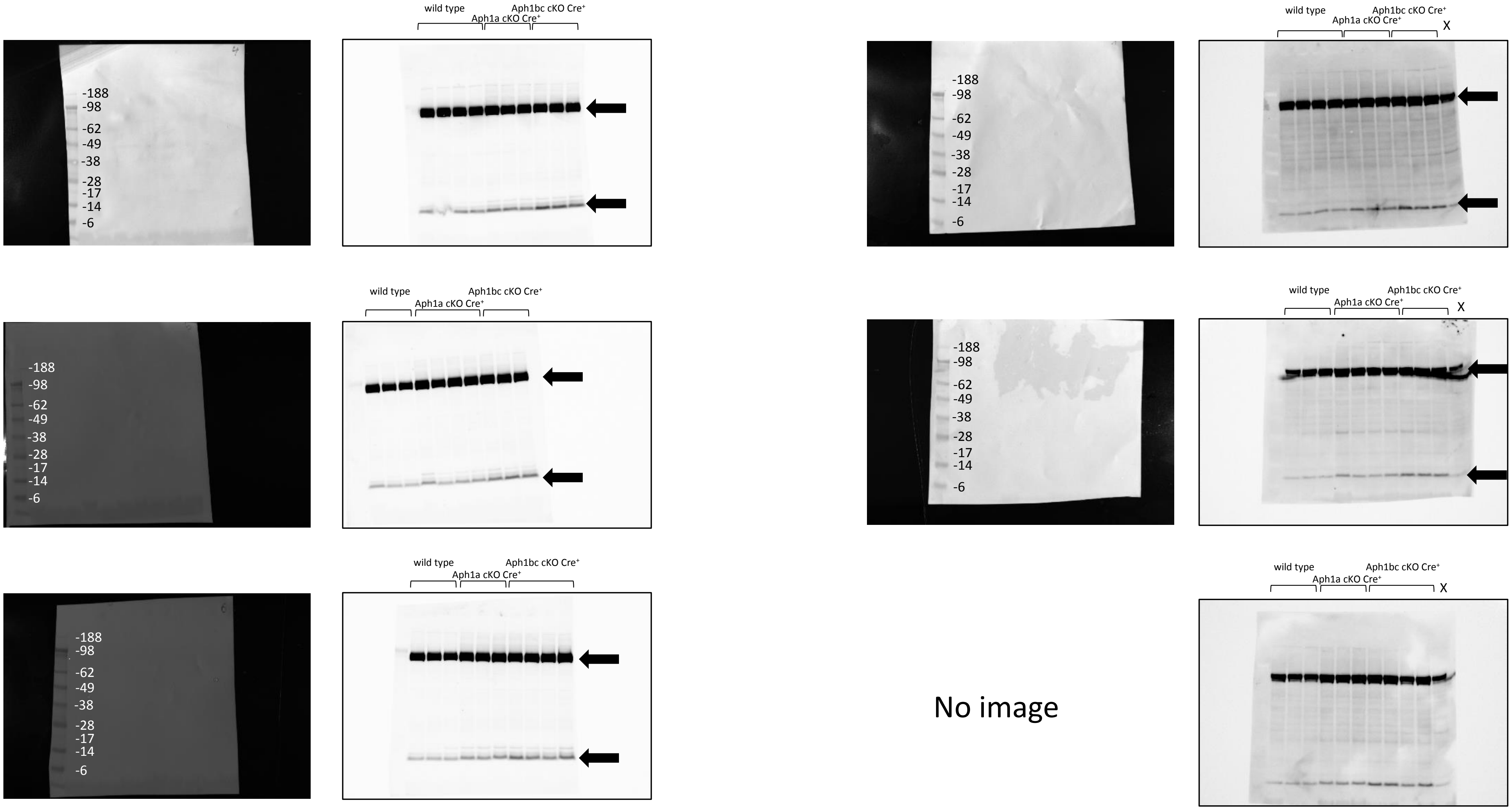

App

Aplp1

Figure 3B

N = 10

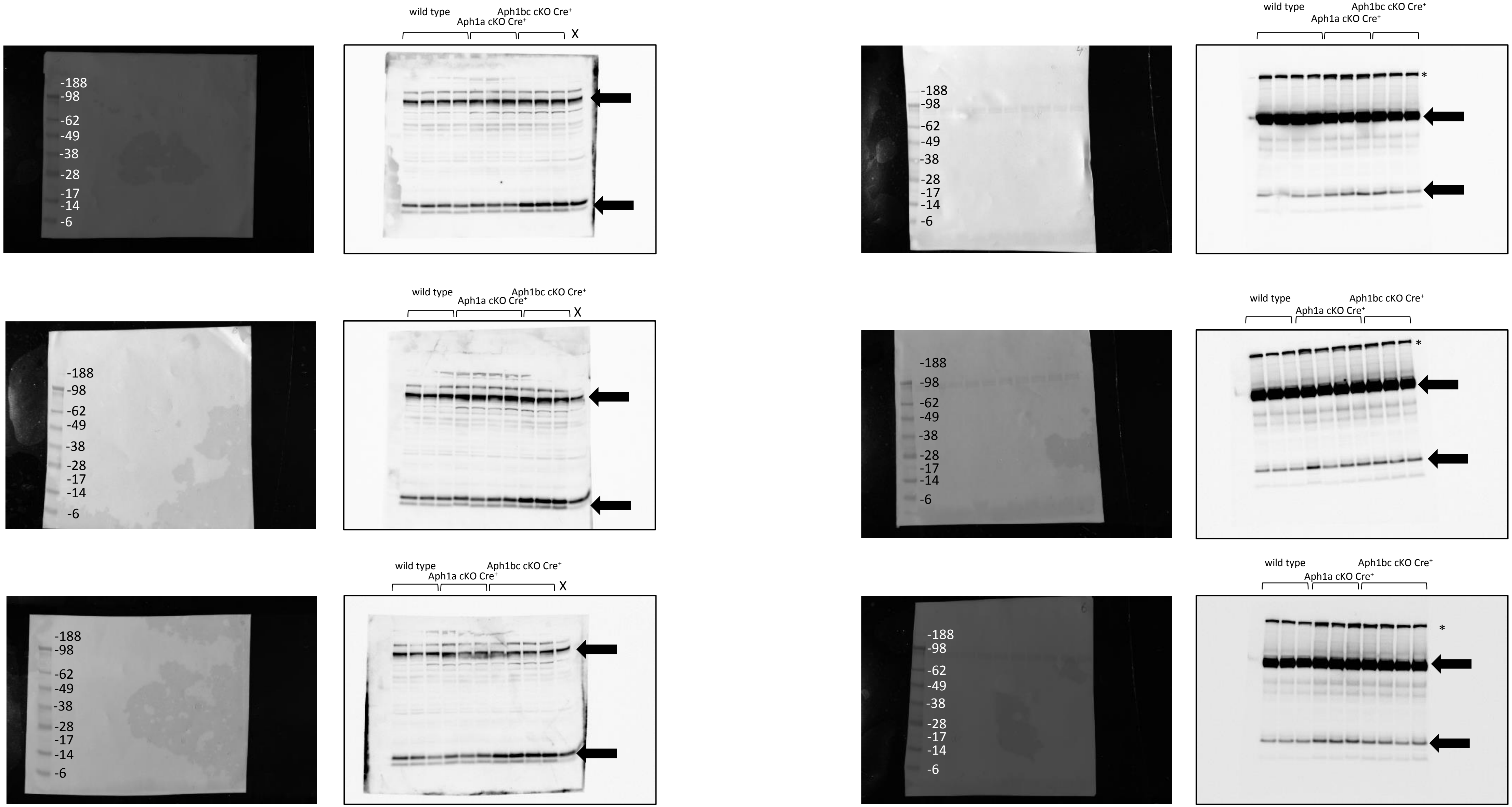

Aplp2

Lrp1

* 600 kDa precuror protein

Figure 3B

N = 10

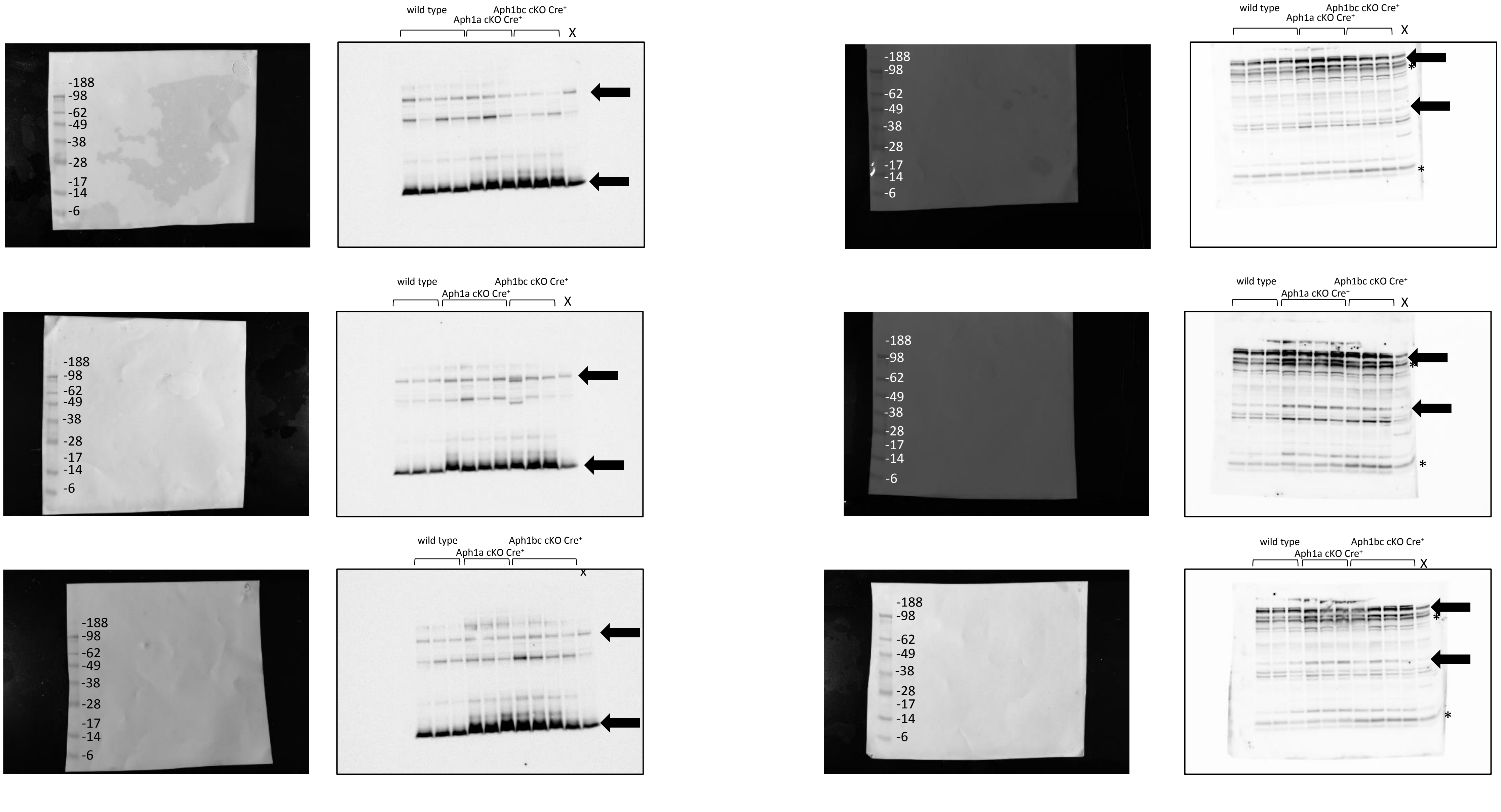

* Signal from previous probing (10 kDa Sdc3)

* Signal from previous probing (Aplp2)

Figure 3B

N = 10

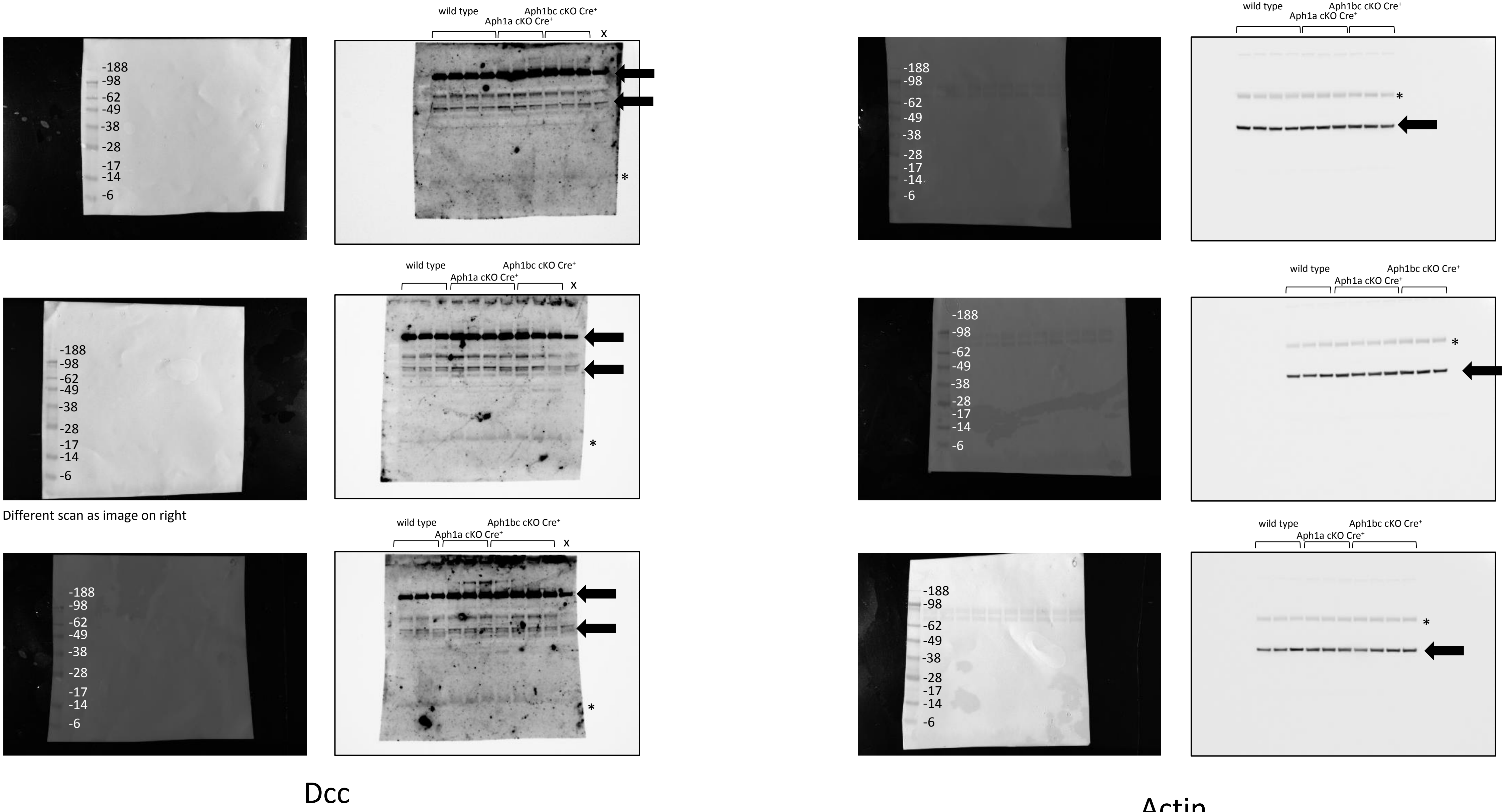

* Signal from previous probing (10 kDa Sdc3)

* Signal from previous probing (80 kDa LRP)
